# First person – John (Eddie) Edmund La Marca

**DOI:** 10.1242/dmm.050179

**Published:** 2023-04-03

## Abstract

First Person is a series of interviews with the first authors of a selection of papers published in Disease Models & Mechanisms, helping researchers promote themselves alongside their papers. John La Marca is first author on ‘ A *Drosophila* chemical screen reveals synergistic effect of MEK and DGKa inhibition in Ras-driven cancer’, published in DMM. John conducted the research described in this article while a research officer in Helena Richardson's lab at La Trobe University, Bundoora, Australia. He is now a research officer in the lab of Gemma Kelly and Marco Herold at the Walter and Eliza Hall Institute of Medical Research, Melbourne, Australia, investigating genetics, cancer and molecular signalling.


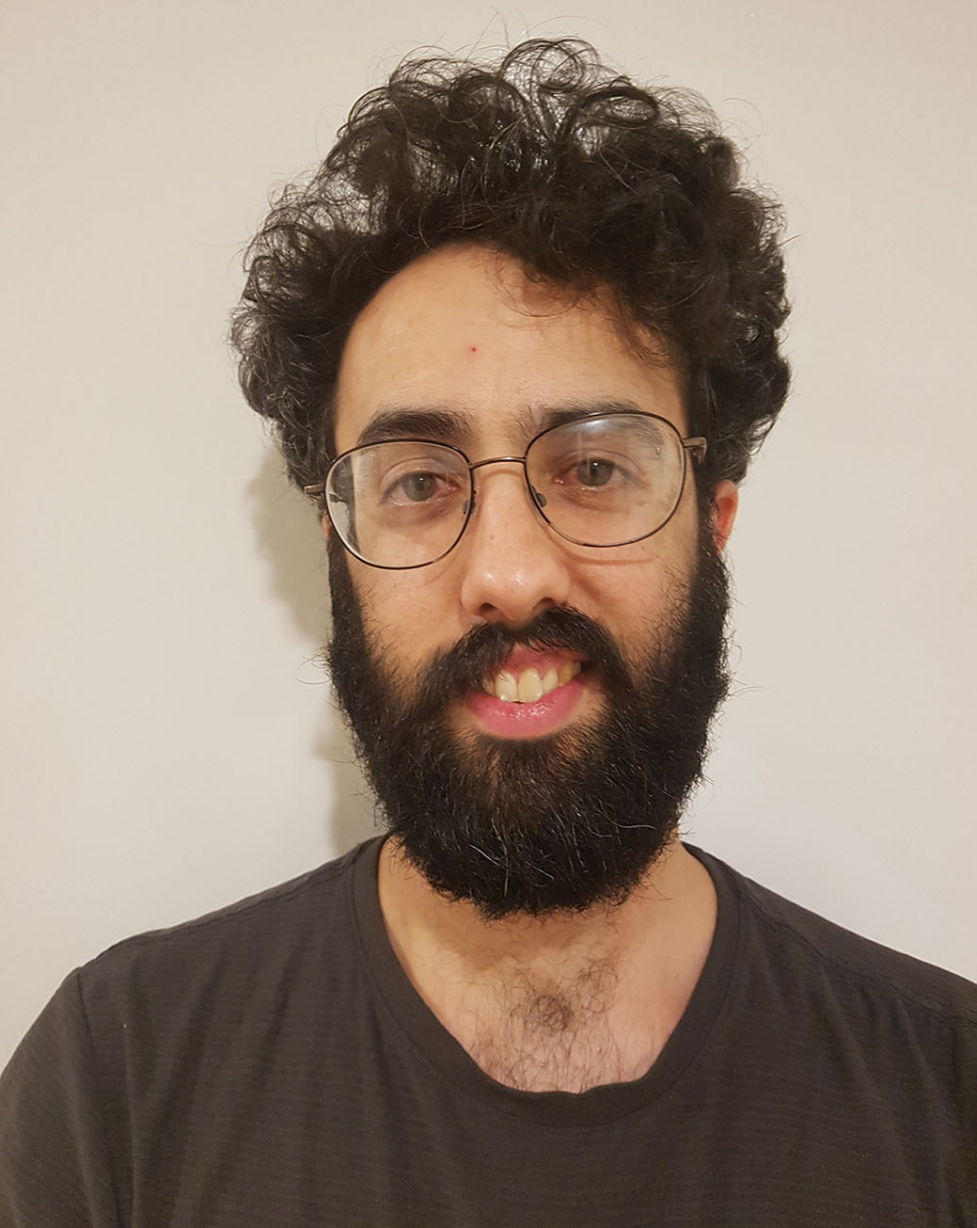


**John La Marca**

**How would you explain the main findings of your paper to non-scientific family and friends?**

Finding new drugs can be difficult, so we try and reuse drugs where possible. One way to figure out if a drug can be repurposed for a different function is to do a ‘screen’, where we test many things very quickly. To make it even quicker, we can use animals other than mice, like insects.

So, in this work, we screened drugs using fruit flies to see whether we could find a drug that might have an anti-cancer role that we didn't previously know about. And we found one called ritanserin that appears to do so.

The fruit flies we used, called *Drosophila melanogaster*, share most of their genes with humans. Because of this similarity, they can be used as what we call a ‘model organism’ and are used to model cancer and, in this case, how those cancers respond to drugs.

**What are the potential implications of these results for your field of research?**

I think we demonstrate, although it doesn't really bear repeating, that *D. melanogaster* persists as a powerful and effective model organism for a huge range of applications, and show a method by which high-throughput drug screens can be conducted in flies, which might be adaptable to other fly-based cancer models. All you really need is a fluorescence-capable microscope.

**What are the main advantages and drawbacks of the experimental system you have used as it relates to the disease you are investigating?**

Of course, the strengths of flies are also balanced by their weaknesses in some areas. While they remain an extremely useful model organism for cancer research, there are limitations in their use. They can't model the disease one-to-one. I have grown to think of their ‘cancers’ more as a collection of phenotypes, which are then relatable to the established hallmarks of cancer. This is still useful, but needs to be complemented with additional data where possible, hence our use of a mammalian cell model of Ras-driven cancer to recapitulate our findings in flies.

**Figure:**
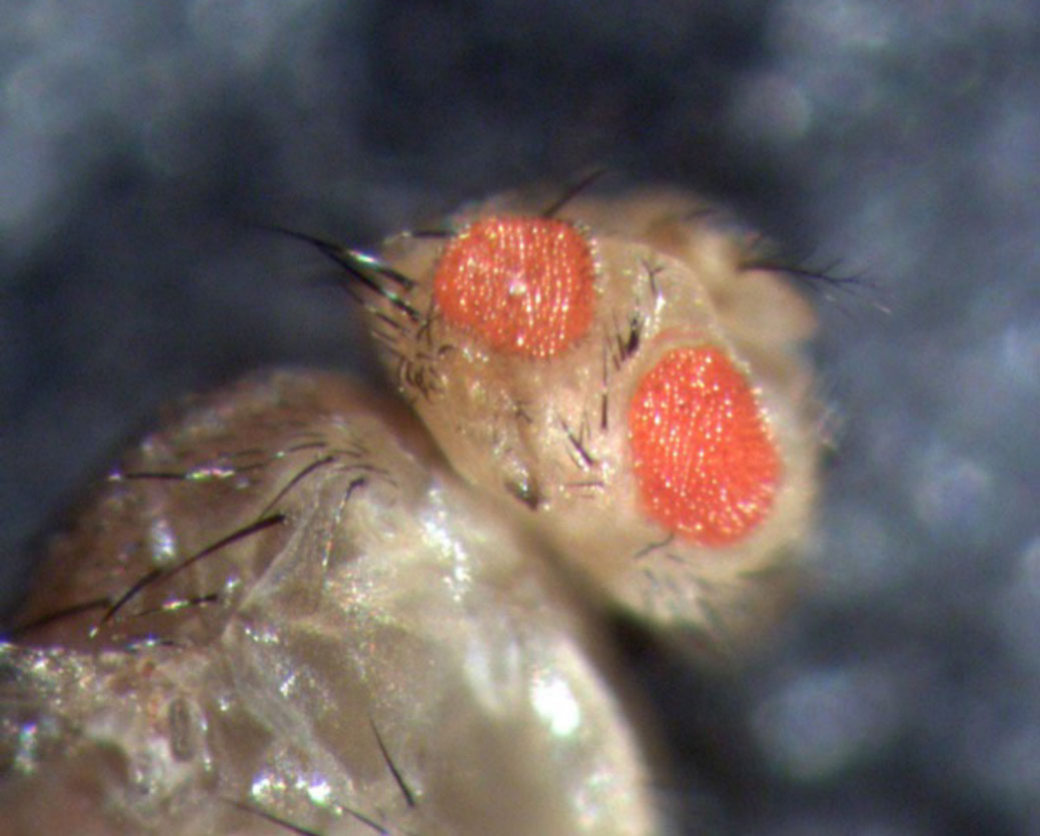
**Parting the red eye: image from a *Drosophila melanogaster* generated as part of a different screen conducted.** Using RNA interference, different genes were knocked down specifically in the eye of the fly. The loss of the *quemao* gene caused quite an interesting phenotype, in which the cells in a horizontal band through the middle of the eye appeared to have either died or never grown, leading to the appearance of the fly having four eyes (two of which are visible here on one side of the fly's head).

**What has surprised you the most while conducting your research?**

I think the most surprising thing was a result that didn't actually make it into the final version of the manuscript. We found that our combination treatment of trametinib and ritanserin was able to quite effectively rescue the larval lethality of the *Ras^V12^/scrib^−^* tumours, with the animals surviving until late pupation. We observed the formation of adult structures in these combination-treated pupae, suggesting that a strong reduction in tumour growth permitted more normal tissue development. However, due to the closure of our fly facility over the course of this study, we were not in a position to complete this experiment with the proper controls (ritanserin alone, specifically). Hopefully this is something that can be investigated in the future in more detail.

“Too many scientists, myself included, are by necessity forced to focus […] on the question of ‘will I even be able to continue in my role as a researcher for another 10 months?’.”

**What do you think is the most significant challenge impacting your research at this time and how will this be addressed over the next 10 years?**

What could it ever be besides funding? Too many scientists, myself included, are by necessity forced to focus less on questions like ‘what is the research challenge for you over the next 10 years?’ and more on the question of ‘will I even be able to continue in my role as a researcher for another 10 months?’. Shifting how research is viewed, particularly basic research, is required: it needs to be seen as less of a political football or punching bag, and more of an investment in the future of our species. It's impossible to predict where the next great discovery will come from, and trying to force it to spring fully formed from an intentionally clinical translation-focused research program is misguided.

**What changes do you think could improve the professional lives of scientists?**

So many problems plague the professional lives of scientists – it would be sensible to consider what aspects are worth saving, before dismantling and rebuilding the entire system from scratch. Were such a thing possible, I think making a system founded on the principle that collaboration should be the driving force behind science, rather than competition, would be an excellent starting point. Most of my best experiences in science have been working in partnership with my peers, and many of the most disheartening parts have been when I've had to compete against them. Science shouldn't be a zero-sum game.

“[…] collaboration should be the driving force behind science, rather than competition […] Science shouldn't be a zero-sum game.”

**What's next for you?**

I've made a huge jump from *Drosophila*-based research into mammalian research, thanks in part to what I learned while working on this manuscript. What's next is just hoping I can hang on in this new role, as it's like starting my PhD all over again in a lot of ways, and there's so much new information to learn. I've never had to worry about things like skeletons or adaptive immune systems before – it's a lot to take in.
